# Comparison of Morphological Characteristics of Antennae and Antennal Sensilla Among Three Species of Gall Wasps (Hymenoptera: Eulophidae)

**DOI:** 10.3390/insects16090976

**Published:** 2025-09-18

**Authors:** Jinting Xie, Yi Liu, Junjue Li, Leming Zhou, Xiu Xu, Zhende Yang

**Affiliations:** Guangxi Colleges and Universities Key Laboratory for Cultivation and Utilization of Subtropical Forest Plantation, Guangxi Key Laboratory of Forest Ecology and Conservation, College of Forestry, Guangxi University, Nanning 530004, China; xiejt333@163.com (J.X.); liuyi202320@163.com (Y.L.); 13978755183@163.com (J.L.); zlm160826@outlook.com (L.Z.); 18791114923@163.com (X.X.)

**Keywords:** antennae, sensilla, SEM, three gall wasps, morphology, differentiation

## Abstract

Various types of sensilla are densely distributed on the antennal surfaces of insects. The specialization of perception and ecological adaptability of antennae in detecting external environmental signals are reflected in the morphology, function, number, and distribution pattern of sensilla. *L. invasa*, *O. bipolaris*, and *O. maskelli* are three types of wasps that harm eucalyptus trees. To investigate the perception and reception methods of chemical signals from eucalyptus trees by the three gall wasps and compare interspecific differences, the antennal morphology and sensillar characteristics of these three gall wasps were systematically analyzed using scanning electron microscopy, including sensillar length, type, distribution, and number. Knee-shaped antennae, comprising the radicle, scape, pedicel, anelli, funicle, and club, are present in all three species. However, significant interspecific differences were observed in total antennal length and funicular number. Five major sensilla categories, comprising nine subtypes, were identified. Among these, five types (BS, CH, CS, TSI, TSII) were common to all three gall wasp species. PSI and PSII were unique to *L. invasa*, while *O. bipolaris* and *O. maskell* shared PSIII. Compared to *O. maskelli*, *O. bipolaris* possessed a significantly greater number of PSIII sensilla, which were also longer. TSIII was found exclusively in *O. bipolaris*. Interspecific differences were evident in antennal morphology, as well as in the number, size, and distribution of the sensilla. These variations in sensilla and antennal shape serve as a morphological foundation for species identification in addition to reflecting ecological adaptation and functional differentiation in environmental signal perception.

## Simple Summary

*Leptocybe invasa*, *Ophelimus bipolaris*, and *Ophelimus maskelli* are three types of gall wasps that harm eucalyptus trees, affecting their growth. To explore the perception and reception mechanisms of chemical signals from eucalyptus trees utilized by gall wasps, scanning electron microscopy was used to conduct a comparative study on the antenna morphology and the length, type, distribution, and number of sensilla of three gall wasps. The antennae of the three species are all knee-shaped, composed of the radicle, scape, pedicel, anelli, funicle, and club. But there were significant interspecific differences in the number of funiculars and antennal length. A total of five types and nine subtypes of sensilla were observed, including Böhm sensilla (BS), Chaetotactic sensilla (CH), Trichoid sensilla (TSI, TSII, TSIII), Placodea sensilla (PSI, PSII, PSIII), and Campaniform sensilla (CS). Among them, PSI and PSII were unique to *L. invasa*, and *O. bipolaris* and *O. maskell* shared PSIII, while TSIII was unique to *O. bipolaris*. The three gall wasps’ antennae exhibit differences in morphology, as well as in the number, size, and distribution of their sensilla. These differences reflect the divergence in sensory functions and ecological adaptations among the three gall wasps, providing morphological evidence for species identification and classification.

## 1. Introduction

Antennae are the primary sensory appendages of insects and play a vital, irreplaceable role in their survival and reproduction [1]. Insects receive various environmental signals through their antennae, which then regulate essential behaviors such as foraging, mating, and avoiding enemies [2]. Different types of sensilla possess specific sensory functions, including chemical sensilla, mechanical sensilla, thermoreceptive sensilla, and olfactory sensilla, among others [3]. Thermoreceptive sensilla respond to temperature changes [4], while mechanical sensilla perceive mechanical stimuli such as vibrations and air currents [5]. Olfactory sensilla play a crucial role in detecting environmental chemical cues (such as pheromones, host odors, and volatile plant materials) [6,7,8]. Previous studies have shown that the types and distribution of sensilla on the antennae are related to the sex of the insects [9,10,11], feeding [12,13], and whether they are sojourned [14,15]. Even within the same species, the types, numbers, and distribution of antennal sensilla can vary [16,17]. The antennae of other insect taxa exhibit various morphologies, with these differences manifesting in the overall structure of the antennae as well as in the function, morphology, number, and arrangement patterns of their sensilla. As essential organs for receiving signals, the antennae have experienced evolutionary pressures and adaptations, which are reflected in these differences [18]. Identifying the antennae and sensilla of insects contributed to clarifying the value of antennal characteristics in biological evolution and the olfactory recognition mechanisms of behavioral control [19].

*Leptocybe invasa* Fisher & La Salle (Hymenoptera: Eulophidae) originated in Australia and is now distributed in the Americas, Africa, Asia, the Middle East, and Europe [20]. By laying eggs on the tender branches, leaf veins, stems, and petioles in various eucalyptus species, *L. invasa* damages them through the formation of typical swollen galls, which cause the leaves and stems to curl and prevent further eucalyptus growth [21]. *Ophelimus maskelli* (Hymenoptera: Eulophidae) forms small, circular, blister-like galls symmetrically on both sides of eucalyptus leaves, leading to extensive leaf drop and significantly impairing eucalyptus growth. This species has been reported in numerous countries worldwide, including the United States [22], Argentina [23], South Africa [24], Tanzania [25], Israel [26], Spain [27], Australia [28], Portugal [29], and China [30]. Moreover, *Ophelimus bipolaris* (Hymenoptera: Eulophidae) causes blister-like galls on eucalyptus leaves, which distort, wilt, and drop the leaves and hinder their normal growth. With the introduction and widespread cultivation of eucalyptus, *O. bipolaris* was first identified in China [31]. *O. maskelli* primarily attacks *Eucalyptus leizhou* No.11, *O. bipolaris* largely infests DH299-5, while *L. invasa* primarily attacks *E. exserta* and *Eucalyptus* clones DH201-2. These three gall wasps have different host preferences.

There have been reports on the antennal sensilla of various Hymenoptera insects, including Braconidae [32], Ichneumonidae [33], Trichogrammatidae [34], Tetrastichidae [35], Pteromalidae [36,37], Agaonidae [38], Eulophidae [39,40,41], Chalcididae [42], Torymidae [43], Megastigmidae [44], Platygastridae [45], Bethylidae [46], Pamphiliidae [47], and Vespidae [48]. However, the antennal sensilla of *O. bipolaris* and *O. maskelli* have not yet been reported. *O. maskelli*, *O. bipolaris*, and *L. invasa* were observed by the authors on the same eucalyptus species, DH288-4, in the experimental area of Dongmen Forest Farm in China. These three insects, which are significant gall-causing eucalyptus pests, have antennal sensilla that are essential for mating, courtship, nest-seeking, and foraging. To investigate how three types of gall wasps perceive and receive chemical signals from eucalyptus trees and to understand the potential mechanisms of these wasps parasitizing the same host eucalyptus tree, scanning electron microscopy observations of the antennal sensilla of adult *O. maskelli*, *O. bipolaris*, and *L. invasa* were conducted. The types, morphologies, and distribution characteristics of antennal sensilla were examined, with differences among them compared. This study provides a reference for future investigations on the host location process of these wasps through comparative analysis.

## 2. Materials and Methods

### 2.1. Insect Sample Collection

The three species of gall wasps used in this study were collected from the same host plant *Eucalyptus* DH288-4, in Dongmen Forestry Center in Guangxi, China (107°90′ E, 22°39′ N). The eucalyptus branches with galls were brought back to the laboratory and placed in a 1000 mL conical flask at room temperature. The flask was filled with water to keep the branches fresh. The branches were covered with sealed nylon mesh bags (15 cm × 30 cm), and the adults that emerged and flew out could be collected in the nylon mesh bags. The emerged adults were collected and stored in the laboratory of the Forestry College, Guangxi University, China (108°29′ E, 22°84′ N). No males were found in the emerged *O. maskelli* and *O. bipolaris*. Therefore, the females were used as the material for the three insects in this study.

### 2.2. Scanning Electron Microscopy

Three samples for each gall wasp were utilized for SEM observations. Firstly, the insect heads were dissected under a stereo microscope (3DM-HK830, Aosvi, Shenzhen, China). The samples were then cleaned using a phosphate-buffered (0.1 mol/L, pH 7.2) solution with an ultrasonic cleaner (AK-031SD, Yuclean, Shenzhen, China) for 5 min to remove impurities. Subsequently, the samples were then transferred to a 2.5% glutaraldehyde solution and fixed for 24 h. Next, the samples were dehydrated sequentially in 75%, 80%, 85%, 90%, and 95% ethanol for 20 s each, followed by drying at room temperature for 12 h. The dried heads were adhered to the sample stage using conductive adhesive and then coated with gold for 1 min. Finally, the prepared samples were scanned and photographed using a scanning electron microscope (FEI Quattro S, Thermo Scientific, Waltham, MA, USA), with an accelerating voltage of 3–5 kV.

### 2.3. Data Processing and Statistical Analysis

The morphology, number, distribution, length, and width of the antennae and sensilla of three gall wasp species were analyzed using a scanning electron microscope. The length and basal width of the antennae were measured with ImageJ 1.8.0 software, and the total number of sensilla on the dorsal and ventral sides of each gall wasp species was counted. Data analysis was conducted using SPSS 26.0 software for ANOVA analysis and independent-samples *t*-tests. Tukey post hoc tests were applied to identify significant differences. Results are presented as mean ± SE. Image processing and graphic creation were conducted using Adobe Photoshop 2023 and Origin 21, respectively.

## 3. Results

### 3.1. Overall Morphology of Antennae

**Figure 1:**
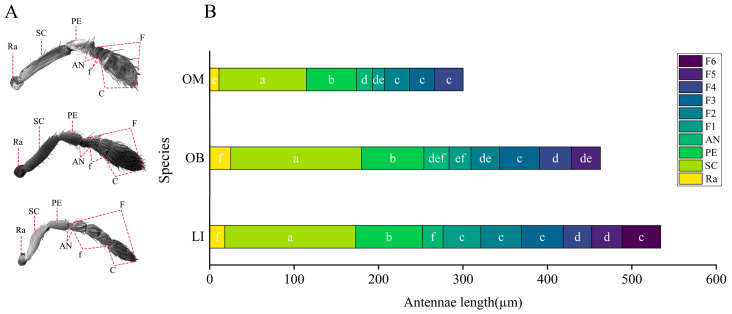
Morphology of the antennae of gall wasps (**A**). The average length of the antennae of gall wasps (**B**). LI: *L. invasa*; OB: *O. bipolaris*; OM: *O. maskelli*; Ra: radicle; SC: scape; PE: pedicel; AN: anelli; f: funicle; C: club; F: flagellum. F1–F6: components of the funicle and club. Lowercase letters a, b, c, etc., indicate significant differences in the length of each antennomere within the same species (*p* < 0.05). In contrast, bars with the same letter indicate no significant difference (*p* > 0.05).

**Figure 2:**
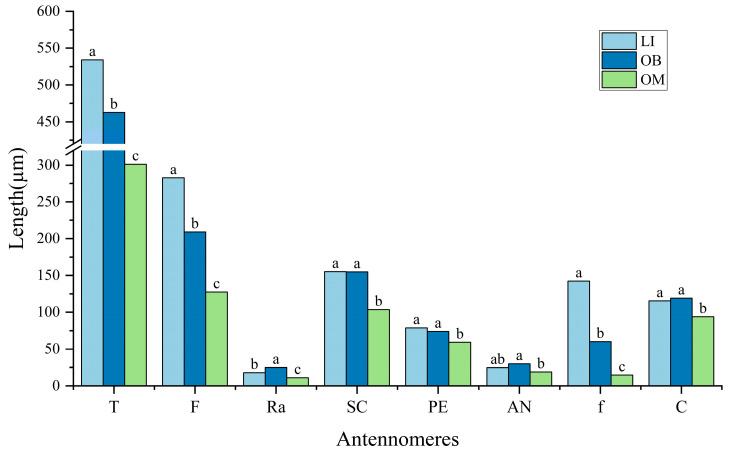
Comparison of the average lengths of each antennomere in different gall wasps. The antennae of different gall wasps and the average length of each antennomere. T: total antennal length; lowercase letters a, b, and c indicate significant differences in the lengths of each antennomere of the same antenna among different gall wasps (*p* < 0.05), while bars sharing the same letter show no significant difference (*p* > 0.05).

The antennae of the three gall wasp species are knee-shaped and located on the lower edge of the compound eyes at the front of the head. The radicle (Ra), scape (SC), pedicel (PE), anelli (AN), funicle (f), and club (C) make up the antennae. The flagellum (F) is made up of the anelli, funicle, and club (Figure 1A). The anelli is made up of four unevenly rounded, disk-shaped parts (Appendix A). *L. invasa* has three funiculars, *O. bipolaris* has two, and *O. maskelli* has one. Three clavomeres are present in all three species. Consequently, the combined number of funiculars and clavomeres for *L. invasa*, *O. bipolaris*, and *O. maskelli* is six, five, and four segments, respectively, designated sequentially as F1–F6. The flagellum is the longest antennal part among the three gall wasps, with lengths ranging from 127.59 ± 0.68 µm to 282.48 ± 4.53 µm. The scape is the second longest, ranging from 103.51 ± 1.66 µm to 155.14 ± 2.42 µm. The pedicel ranges from 59.23 ± 2.31 µm to 78.81 ± 2.66 µm, and the radicle ranges from 10.96 ± 0.76 µm to 24.80 ± 1.75 µm. Further analysis of the lengths of each component within the same species shows that the scape is the longest antennomere, significantly longer than the others, and the pedicel is the second longest, while the radicle is the shortest (*p* < 0.05; Figure 1B). The average total antennal length of the three gall wasps differs significantly from one another, according to statistical analysis (*p* < 0.05; Figure 2). *L. invasa* has the longest antennae, measuring 534.24 ± 0.68 µm, followed by *O. bipolaris* at 462.73 ± 3.00 µm, and *O. maskelli* has the shortest at 301.29 ± 0.68 µm. Comparing the lengths of the same antennal parts among the species, significant differences exist in the lengths of the radicle, funicle, and flagellum (*p* < 0.05). *O. bipolaris* has the longest radicle, followed by *L. invasa*, with *O. maskelli* having the shortest. Regarding the funicle and flagellum lengths, *L. invasa* is the longest, followed by *O. bipolaris*, and *O. maskelli* is the shortest. The variation in flagellum length is attributed to the differing numbers of funiculars. Additionally, the lengths of the scape, pedicel, and club in *O. maskelli* are significantly shorter than those in *L. invasa* and *O. bipolaris* (*p* < 0.05). However, for these antennomeres, no significant differences are observed between *L. invasa* and *O. bipolaris* (*p* > 0.05). The length of the anelli in *L. invasa* does not differ significantly from that in *O. maskelli* and *O. bipolaris* (*p* > 0.05). Still, the length of the anelli in *O. maskelli* is significantly shorter than that of *O. bipolaris* (*p* < 0.05).

### 3.2. Antennal Sensilla

**Figure 3:**
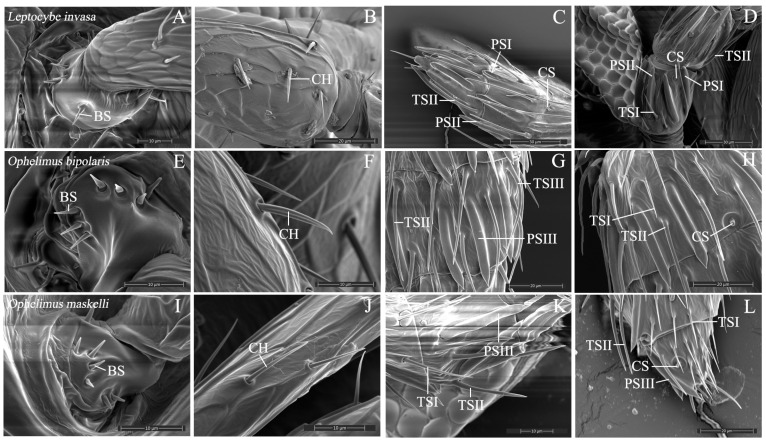
SEM images of gall wasps’ sensilla.The sensilla of *L. invasa* (**A**–**D**); The sensilla of *O. bipolaris* (**E**–**H**); The sensilla of *O. maskelli* (**I**–**L**). BS: Böhm sensilla; CH: chaetica sensilla; CS: campaniform sensilla; PSI: placodea sensilla I; PSII: placodea sensilla II; PSIII: placodea sensilla III; TSI: trichodea sensilla I; TSII: trichodea sensilla II; TSIII: trichodea sensilla III.

Five types and nine subtypes of sensilla were identified on the antennae, including Böhm sensilla (BS), Chaetic sensilla (CH), Campaniform sensilla (CS), Placodea sensilla (PSI, PSII, PSIII), and Trichodea sensilla (TSI, TSII, TSIII) (Figure 3). Seven sensilla types, BS, CH, CS, PSI, PSII, TSI, and TSII, are found in *L. invasa*. BS, CH, CS, PSIII, TSI, TSII, and TSIII are the seven sensilla types found in *O. bipolaris*, with BS, CH, CS, PSIII, TSI, and TSII being the six sensilla types found in *O. maskelli*. The morphological and size characteristics of the sensilla are illustrated in Figure 3 and summarized in Table 1.

#### 3.2.1. Böhm Sensilla

The Böhm sensillum (BS) is located on the radicle (Figure 3A,E,I). Its surface is nearly smooth, nearly perpendicular to the surface, and resembles a short spine. BS was found in all three gall wasp species. Its dimensions are 3.03 ± 0.31 µm to 5.78 ± 0.39 µm for length and 0.71 ± 0.04 µm to 1.26 ± 0.05 µm for width. Interspecific comparisons showed that the BS of *L. invasa* is the longest, considerably longer than those of *O. bipolaris* and *O. maskelli* (*p* < 0.05). While *O. bipolaris* and *L. invasa* both have significantly broader BS than *O. maskelli* (*p* < 0.05), *O. bipolaris*’s BS is the widest and does not differ considerably from *L. invasa*’s (*p* > 0.05). Within the same gall wasp species, BS is significantly shorter than CH, TS, and PS, and markedly narrower than PS (*p* < 0.05). The BS of *L. invasa* is slender and longer, that of *O. bipolaris* is short and stubby, and that of *O. maskelli* is relatively thin and short.

#### 3.2.2. Chaetica Sensilla

The Chaetica sensillum (CH) is illustrated in Figure 3B,F,J. On the antennal surface, it is upright and spine-like, with a broad base that tapers toward the tip. All three of the gall wasp species under study have the CH. Its length ranges from 12.25 ± 0.44 µm to 14.66 ± 0.45 µm, and its width varies from 0.98 ± 0.02 µm to 1.12 ± 0.02 µm. *O. bipolaris* has the longest CH, significantly longer than that of *L. invasa*, while *L. invasa* has the shortest CH (*p* < 0.05). *L. invasa* exhibits the widest CH, followed by *O. bipolaris*; both are significantly wider than those of *O. maskelli* (*p* < 0.05). Within each gall wasp species, the length of the CH differs significantly from the lengths and widths of other sensillum types. In all three species, the CH is substantially longer than the BS and CS, and it is considerably narrower than the PS in terms of width (*p* < 0.05).

#### 3.2.3. Trichodea Sensilla

Trichodea sensilla (TS) is illustrated in Figure 3C,D,G,H,K,L. Three types of TS were identified across three gall wasp species: TSI and TSII were common to all three species, while TSIII was unique to O. bipolaris. TSI are inclined approximately 30°–45° relative to the antennal axis, with a pointed tip directed toward the antennal apex. It was discovered on the funicle and club of all three species, as well as the anelli of *O. maskelli* and *O. bipolaris*. TSI length ranges from 13.69 ± 0.12 µm to 21.30 ± 0.75 µm, and width ranges from 0.97 ± 0.07 µm to 1.36 ± 0.07 µm. TSI length differs significantly among the three species, with *L. invasa* having significantly longer TSI than *O. bipolaris* and *O. maskelli* (*p* < 0.05). Regarding TSI width, *O. maskelli* is considerably narrower than *O. bipolaris* (*p* < 0.05). TSII has a pointed tip similar to TSI but is longer and wider. TSII length ranges from 25.02 ± 0.86 µm to 50.84 ± 0.97 µm, and width ranges from 1.62 ± 0.15 µm to 2.00 ± 0.04 µm. TSII length also differs significantly among the three species, with *L. invasa* having significantly longer TSII than *O. bipolaris* and *O. maskelli* (*p* < 0.05). Within the same species, TSII length varies noticeably from other sensillum types. TSII is the longest sensillum type in both *L. invasa* and *O. bipolaris* (*p* < 0.05). In *O. maskelli*, it is significantly shorter than PSIII but is the second-longest sensillum type in this species (*p* < 0.05). TSIII, unique to *O. bipolaris*, which tapers from base to apex and has a small curvature from middle to tip. It measures 33.86 ± 0.50 µm in length and 1.87 ± 0.06 µm in width.

#### 3.2.4. Placodea Sensilla

The Placodea sensillum (PS) is illustrated in Figure 3C,D,G,K. Three subtypes (PSI, PSII, PSIII) were identified. PSI and PSII were discovered in funiculars and clavomeres of *L. invasa*. The PSI type has a rounded, short, and narrow front end, almost entirely attached to the surface of the antenna, or only the tip is free. The length of PSI is 32.73 ± 0.17 µm, and its width is 5.12 ± 0.13 µm. PSII is relatively elongated, with the tip being sharper than that of PSI. The base is attached to the surface of the antenna for 1/2 to 2/3 of its length, while the remaining part is detached from the antenna surface, with the front end being sharp. PSII measures 36.26 ± 0.14 µm in length and 2.59 ± 0.07 µm in width. PSIII was found on the funiculars and clavomeres of *O. bipolaris*, as well as on the clavomeres of *O. maskelli*. Morphologically, it is similar to PSII but shorter. The basal 3/4 to 5/6 is attached to the surface, while the distal antennomere remains free, terminating in a pointed apex. PSIII length ranges from 27.42 ± 0.73 µm to 34.24 ± 0.12 µm, and width ranges from 2.49 ± 0.09 µm to 3.47 ± 0.06 µm. Significant differences in both length and width of PSIII were found between *O. bipolaris* and *O. maskelli*, with *O. bipolaris* having longer and wider PSIII (*p* < 0.05). Among the PS subtypes in *L. invasa*, PSII is the second longest, significantly longer than other sensilla within this species (*p* < 0.05), and PSI is the widest, considerably wider than other sensilla in *L. invasa* (*p* < 0.05).

#### 3.2.5. Campaniform Sensilla

The Campaniform sensillum (CS) is illustrated in Figure 3C,D,H,L. It is a dome-shaped protrusion with a bluntly rounded tip, located within a circular depression, and is oriented nearly perpendicular to the antennal surface. Its length ranges from 2.51 ± 0.33 µm to 2.77 ± 0.06 µm, and its width varies from 1.15 ± 0.04 µm to 1.37 ± 0.08 µm. Among the species examined, *L. invasa* exhibits the broadest distribution, followed by *O. bipolaris*, with *O. maskelli* being the least widespread. No significant differences were observed in the length and width of the CS among the three gall wasp species (*p* > 0.05). However, within individual gall wasp species, the CS length in *L. invasa* and *O. bipolaris* was significantly shorter compared to other sensory types (*p* < 0.05), resulting in the shortest CS lengths across all sensory types within these two species.

#### 3.2.6. Number and Distribution of Sensilla

**Figure 4:**
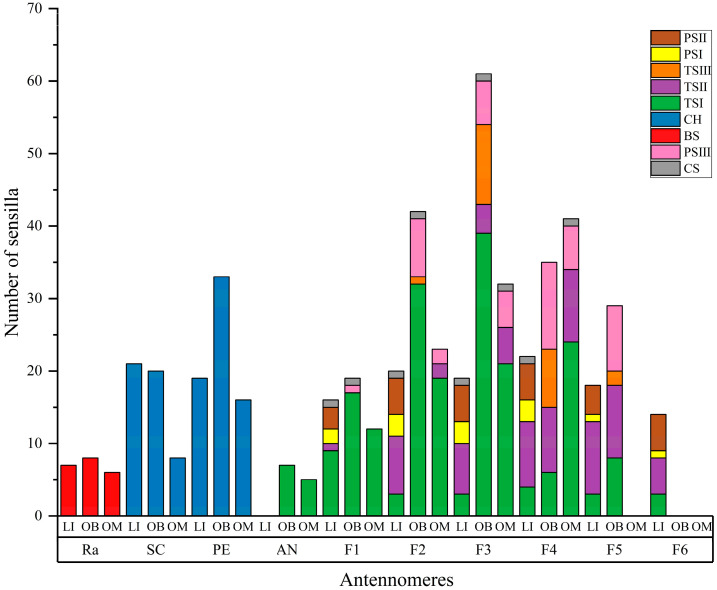
Distribution of Gall Wasp Sensilla.

The number and distribution of sensilla in the three gall wasp species are presented in Table 2 and Figure 4. BS was consistently located on the radicle in all species, with counts ranging from 6 to 9. Notably, *O. bipolaris* has a higher number of BS compared to *O. maskelli* (*p* < 0.05). Chaetica sensilla, found on the pedicel and scape, demonstrated significant interspecific differences: *O. bipolaris* possessed the greatest abundance, followed by *L. invasa*, while *O. maskelli* had the fewest (*p* < 0.05). TSI was distributed on the flagellum of *O. maskelli* and *O. bipolaris*, and the funiculars and clavomeres of *L. invasa*, with *O. bipolaris* exhibiting significantly more TSI than the other two species (*p* < 0.05). The distribution of TSII varied among species: in L. invasa, TSII was present on antennomere F1–F6; in *O. bipolaris*, it was confined to antennomere F3–F5; and in *O. maskelli*, it occurred on antennomere F2–F4. This corresponds to the entire funiculars and clavomeres in *L. invasa*, and the clavomeres in both *O. maskelli* and *O. bipolaris*. Statistical analysis revealed that *L. invasa* had significantly more TSII than *O. bipolaris* and *O. maskelli* (*p* < 0.05). It is worth noting that TSIII is unique to *O. bipolaris*, localized solely on antennomere F2–F5. Regarding placoid sensilla, PSI and PSII were restricted to antennomere F1–F6 in *L. invasa*. PSIII was distributed on antennomere F1–F5 in *O. bipolaris* and F2–F4 in *O. maskelli*, with *O. bipolaris* exhibiting a significantly higher PSIII count than *O. maskelli* (*p* < 0.05). CS was sparsely distributed across the funiculars and clavomeres in all three species, with counts ranging from 1 to 4; *L. invasa* had the highest CS abundance, followed by *O. bipolaris*, and *O. maskelli* had the lowest. Within *L. invasa*, CH and TSII were significantly more abundant than other sensillum types (*p* < 0.05). In both *O. bipolaris* and *O. maskelli*, TSI significantly outnumbered other types, making it the most abundant sensillum (*p* < 0.05); CS was the least abundant sensillum type across all three gall wasp species.

## 4. Discussions

### 4.1. Antennal Morphological Differentiation

The antennae of the three gall wasp species examined exhibit a knee-shaped configuration, comprising a scape, pedicel, and flagellum. This conserved basic morphology and positioning align with previous findings on antennal structures in Hymenoptera [49,50,51]. Nonetheless, notable interspecific differences were identified in the number of funiculars, overall antennal length, and the dimensions of individual antennomeres. Such variation in funicular number is likely linked to the sensilla functions of gall wasps. In this study, differences in funicular count corresponded with variations in both the number and length of flagellomeres. Their capacity to detect long-range chemical signals may be enhanced by this structural variety, enabling them to adapt to more extensive host-searching needs [52]. Within gender and among species, the flagellum showed the most variation in length and shape [5]. Previous studies have shown that a longer flagellum has a larger surface area, which enables it to support more sensilla and enhances the effectiveness of volatile ambient chemical detection [53,54,55]. In this case, *L. invasa*’s longest flagellum matches its largest overall antennal length, which may help explain its greater host range or the requirement for long-distance chemical signal detection. Conversely, the short flagellum of *O. maskelli* may be adapted for perceiving chemical or mechanical signals at close range within specific microhabitats, reflecting an evolutionary trade-off between energy allocation and functional demands. The extended funicle in *L. invasa* increases the sensilla-bearing surface area, thereby enhancing sensitivity to host volatiles; this finding aligns with Xue S’s research on *Andricus moriokae*, which underscores the influence of flagellum segmentation and sensilla density on host location efficacy [56]. Furthermore, interspecific variation in antennomere length reflects morphological divergence among gall wasp species. The scape is essential for supporting and facilitating antennal movement [57], and an elongated scape may enhance the mechanical efficiency of antennal motions, allowing for more flexible spatial exploration. Notably, the significantly shorter scape in *O. maskelli* may be related to microhabitat adaptation, a phenomenon also reported in parasitoid wasps. For instance, the antennal movement range of *Aphidius gifuensis* correlates directly with host microhabitat size, and cold storage alters its antennal and sensillar morphology [58]. At both phylogenetic and taxonomic levels, these antennal morphological variations constitute critical characters for species identification and classification. The observed differences enrich the suite of morphological indices employed in gall wasp species taxonomy. Specifically, variation in funicular number and antennomere lengths serves as valuable supplementary traits for discriminating among the three species studied, thereby aiding more accurate species identification and phylogenetic analysis.

### 4.2. Diversity and Functional Specialization of Sensilla

Different types of antennal sensilla serve distinct functions in insect perception. This study identified five major types (Böhm sensilla, Chaetica sensilla, Campaniform sensilla, Placodea sensilla, and Trichodea sensilla) with nine subtypes, consistent with those reported in other Hymenoptera, indicating a conserved sensilla organ structure within this order. However, variations in sensillar type, morphology, and distribution among the three gall wasp species suggest adaptive divergence. Böhm sensilla, which serve as proprioceptors at antennal joints, may play a crucial role in the precise control of antennal movement [59,60]. Their consistent distribution on the radicle, particularly at intersegmental junctions, suggests functions in perceiving mechanical stimuli, detecting antennal position, and coordinating motion [61,62]. Chaetica sensilla detect mechanical stimuli; some studies indicate they may also perceive chemical cues and selectively respond to mechanical disturbances, as demonstrated in *Holcocerus hippophaecolus* [63]. The longest CH in *O. bipolaris* may enhance tactile discrimination on rough surfaces, while the broader CH in *L. invasa* could increase sensitivity to low-frequency vibrations. In addition, some scholars have speculated in their research on *Pissodes nitidus* that the CH are chemical sensilla performing olfactory functions, primarily related to the female search for oviposition sites by detecting the volatile chemicals emitted by host plants [64]. During mating, when males tap females with their antennae, CH may decode species-specific vibrational signals. Trichodea sensilla are primarily distributed on the flagellum and likely function as mechanoreceptors as well as basic olfactory sensilla for detecting sex pheromones and host volatiles [65,66,67]. Studies on *Bombyx mori* and *Helicoverpa armigera* confirm their critical role in pheromone detection [68,69]. Subtype differentiation (categorized as TSI, TSII, and TSIII) and the distribution patterns of these subtypes reflect the specificity of chemical perception. In the case of *L. invasa*, the relatively longer TSI and TSII structures may play a crucial role in enhancing the adsorption of hydrophobic odor molecules, which aids host recognition in complex environments [65]. TSIII, unique to *O. bipolaris* and restricted to specific antennomeres, likely evolved to detect host-specific volatiles, a result of long-term coevolution. Placodea sensilla are the most common olfactory sensilla in Hymenoptera and are essential for detecting long-range volatile compounds [68]. Electrophysiological studies have confirmed their concentration-dependent responsiveness to plant volatiles [70]. In this study, the morphology of PS showed the most significant divergence: *L. invasa* possesses subtypes PSI and PSII, whereas *O. bipolaris* and *O. maskelli* have only PSIII. As key olfactory receptors, PS vary in length, number, arrangement, and degree of separation [71], which increases the air-exposed surface area to capture more odor molecules [72]. PSII, predominant in *L. invasa* and attached to the base by only 1/2 to 2/3 of its base, which maximizes air contact for efficient adsorption of lipophilic pheromones. Conversely, PSIII in *O. maskelli* is likely adapted for short-range signal detection. The simultaneous presence of these three wasp species on the same host may be attributed to the differentiation of their sensilla functions. For example, *L. invasa*’s broad-spectrum sensilla system allows it to occupy competitive niches such as tender branches, leaf veins, and stems. *O. maskelli* and *O. bipolaris* avoid direct competition through specialized sensilla, exploiting microhabitats on the upper and lower leaf surfaces. Similar niche partitioning occurs in fig wasps (Agaonidae), where coexisting genera (*Sycoscapter*, *Philotrypesis*, and *Watshamiella*) exhibit significant differences in ovipositor length, enabling egg deposition into distinct fig tissue layers or developmental stages [73,74].

## 5. Conclusions

In this study, antennal morphology and sensillar characteristics were systematically analyzed for three gall wasp species (*L. invasa*, *O. maskelli*, *O. bipolaris*). Significant interspecific differences were observed morphologically in funicular number, total antennal length, and individual antennomere lengths. *L. invasa* exhibited the longest antennae with three funiculars, whereas *O. maskelli* possessed the shortest antennae with only one funicular. Sensillar traits (type, number, distribution, size) also demonstrated species-specific variation. The antennae of all three species exhibit a knee-shaped structure composed of the radicle, scape, pedicel, anelli, funicle, and club. Five major sensilla types (BS, CH, CS, TS, PS), comprising nine subtypes, were identified and were common across all species. *L. invasa* was characterized by seven sensilla types, including unique PSI and PSII distributed throughout the flagellum. *O. bipolaris* also possessed seven types, featuring unique TSIII concentrated on antennomere F2–F5. *O. maskelli* exhibited six types, with PSIII significantly less numerous and smaller than in *O. bipolaris*. Quantitative and distributional analyses further highlighted key differences: *O. bipolaris* displayed the highest number of TSI, while *L. invasa* exhibited the longest TSII and broadest CS distribution. These differences not only reflect functional and ecological adaptations in sensory perception among the three species, driving their niche separation on the same host plant, but also provide morphological evidence for the identification and classification of this group. Future multidisciplinary approaches (e.g., molecular biology, electrophysiology, and behavior associations) are suggested to explore the sensory function mechanisms, ecological associations, and evolutionary drivers underlying these antennal differences.

## Figures and Tables

**Table 1:** Length and Width of Sensilla (µm).

Castes and Types	Length			Width		
	LI	OB	OM	LI	OB	OM
BS	5.78 ± 0.39 Af	3.13 ± 0.22 Be	3.03 ± 0.31 Bd	1.20 ± 0.08 Ad	1.26 ± 0.05 Acd	0.71 ± 0.04 Bd
CH	12.25 ± 0.44 Be	14.66 ± 0.45 Ad	13.19 ± 0.71 ABc	1.12 ± 0.02 Ad	1.10 ± 0.01 Ad	0.98 ± 0.02 Bc
TSI	21.30 ± 0.75 Ad	16.97 ± 0.16 Bc	13.69 ± 0.12 Cc	1.24 ± 0.09 ABcd	1.36 ± 0.07 Ac	0.97 ± 0.07 Bc
TSII	50.84 ± 0.97 Aa	35.57 ± 0.18 Ba	25.02 ± 0.86 Cb	1.62 ± 0.15 Ac	2.00 ± 0.04 Ab	1.98 ± 0.05 Ab
TSIII	-	33.86 ± 0.50 b	-	-	1.87 ± 0.06 b	-
PSI	32.73 ± 0.17 c	-	-	5.12 ± 0.13 a	-	-
PSII	36.26 ± 0.14 b	-	-	2.59 ± 0.07 b	-	-
PSIII	-	34.24 ± 0.12 Ab	27.42 ± 0.73 Ba	-	3.47 ± 0.06 Aa	2.49 ± 0.09 Ba
CS	2.77 ± 0.06 Ag	2.51 ± 0.33 Ae	2.69 ± 0.09 Ad	1.25 ± 0.10 Acd	1.37 ± 0.08 Ac	1.15 ± 0.04 Ac

Note: Capital letters A, B, C denote differences among the same antenna sensilla across different gall wasps, while lowercase letters a, b, c, etc., indicate differences in the lengths and widths of the different antennal sensilla of the same gall wasp (*p* < 0.05). The same letters indicate insignificant differences (*p* > 0.05). Values are presented as the means ± standard errors.

**Table 2:** Number of Gall Wasp Sensilla.

Castes and Types	Number		
	LI	OB	OM
BS	7.33 ± 0.88 Ad	8.67 ± 0.88 Ae	6.67 ± 1.20 Ad
CH	40.67 ± 1.45 Ba	53.33 ± 1.20 Ab	24.00 ± 1.15 Cb
TSI	25.67 ± 1.20 Cb	109.33 ± 0.88 Aa	81.00 ± 1.53 Ba
TSII	40.00 ± 1.15 Aa	23.00 ± 0.58 Bd	17.67 ± 0.88 Cc
TSIII	-	22.67 ± 1.15 d	-
PSI	13.33 ± 1.20 c	-	-
PSII	27.67 ± 0.88 b	-	-
PSIII	-	36.67 ± 0.88 Ac	13.66 ± 1.20 Bc
CS	4.00 ± 0.58 Ad	3.00 ± 0.58 Af	1.66 ± 0.67 Ad

Note: The capital letters A, B, C represent the differences in the number of the same antennae among different gall wasps (*p* < 0.05), while the lowercase letters a, b, c, etc., represent the differences in the number of different antennae among the same gall wasps (*p* < 0.05). The same letters indicate an insignificant difference (*p* > 0.05). The values shown are the mean ± standard error.

**Figure A1:**
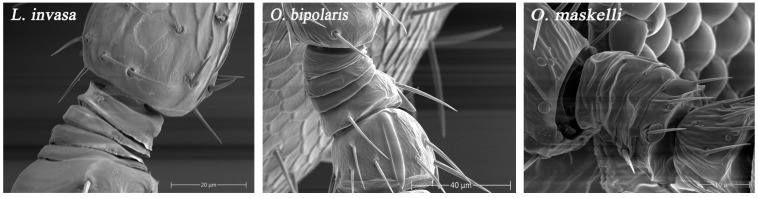
The Anelli of Three Gall Wasps.

## Data Availability

The data presented in this study are available in the article.

## References

[1] Wechsler S.P., Bhandawat V. (2023). Behavioral algorithms and neural mechanisms underlying odor-modulated locomotion in insects. J. Exp. Biol..

[2] Gadenne C., Barrozo R.B., Anton S. (2016). Plasticity in insect olfaction: To smell or not to smell?. Ann. Rev. Entomol..

[3] Lan X.N., Xiang S.S., Zhu H. (2023). Research progress on the types and functions of insect antennae sensilla. J. Environ. Entomol..

[4] Yokohari F. (1981). The sensillum capitulum, an antennal hygro-and thermoreceptive sensillum of the cockroach, *Periplaneta americana* L. Cell Tissue Res..

[5] Schneider D. (1964). Insect antennae. Ann. Rev. Entomol..

[6] Lopes O., Barata E.N., Mustaparta H., Araújo J. (2002). Fine structure of antennal sensilla basiconica and their detection of plant volatiles in the eucalyptus woodborer, *Phoracantha semipunctata* Fabricius (Coleoptera: Cerambycidae). Arthropod Struct. Dev..

[7] Gill K.P., Wilgenburg E.V., Macmillan D.L., Elgar M.A. (2013). Density of antennal sensilla influences efficacy of communication in a social insect. Am. Nat..

[8] Xu M., Guo H., Hou C., Wu H., Huang L.Q., Wang C.Z. (2016). Olfactory perception and behavioral effects of sex pheromone gland components in *Helicoverpa armigera* and *Helicoverpa assulta*. Sci. Rep..

[9] Babu M.J. (2019). Antennal sensilla of the weaver ant *Oecophylla smaragdina* (F.)-males and females sense differently?. Indian J. Entomol..

[10] Fialho M.D.Q., Guss-Matiello C.P., Zanuncio J.C., Campos L.A.O., Serrao J.E. (2014). A comparative study of the antennal sensilla in corbiculate bees. J. Apic. Res..

[11] Xu W.T., Liu G.T., Wang Q.K., Yan L.P., Liu X.H., Li X.Y., Pape T., Zhang D. (2022). Ultrastructure of antennal sensory organs in nine flesh flies (Diptera: Sarcophagidae): New insight into the definition of family Sarcophagidae. Insects.

[12] Polidori C., Jorge A., Ornosa C. (2020). Antennal morphology and sensillar equipment vary with pollen diet specialization in *Andrena* bees. Arthropod Struct. Dev..

[13] Polidori C., Jorge G.A., Nieves-Aldrey J.L. (2012). Antennal sensillar equipment in closely related predatory wasp species (Hymenoptera: Philanthinae) hunting for different prey types. Comptes Rendus Biol..

[14] Galvani G.L., Gonzalez-Vaquero R.A., Guerra-Navarro C., Settembrini B.P. (2017). Antennal sensilla of cleptoparasitic and non-parasitic bees in two subfamilies of Apidae. Apidologie.

[15] Wcislo W.T. (1995). Sensilla numbers and antennal morphology of parasitic and non-parasitic bees (Hymenoptera: Apoidea). Int. J. Insect Morphol. Embryol..

[16] Agren L., Hallberg E. (1996). Flagellar sensilla of bumble bee males (Hymenoptera, Apidae, Bombus). Apidologie.

[17] Schwartz H.S., Preisler H.D., Kanter P.M. (1981). DNA damage in AML cell exposed to adriamycin; correlations with clinical response to therapy. Leuk. Res..

[18] Elgar M.A., Zhang D., Wang Q. (2018). Focus: Ecology and evolution: Insect antennal morphology: The evolution of diverse solutions to odorant perception. Yale J. Biol. Med..

[19] Nemeth D.C., Ammagarahalli B., Layne J.E., Rollmann S.M. (2018). Evolution of coeloconic sensilla in the peripheral olfactory system of *Drosophila mojavensis*. J. Insect Physiol..

[20] Dittrich S.G., Wingfield M.J., Hurley B.P., Slippers B. (2012). Diversity in *Eucalyptus* susceptibility to the gall-forming wasp *Leptocybe invasa*. Agric. For. Entomol..

[21] Huang Z.Y., Li J., Lu W., Zheng X.L., Yang Z.D. (2018). Parasitoids of the eucalyptus gall wasp *Leptocybe* spp.: A global review. Environ. Sci. Pollut. Res..

[22] Burks R.A., Mottern J.L., Pownall N.G., Paine D.T. (2015). First record of *Closterocerus chamaeleon*, parasitoid of the Eucalyptus gall wasp *Ophelimus maskelli* (Hymenoptera, Chalcidoidea, Eulophidae), in the New World. ZooKeys.

[23] Aquino D.A., Hernández C.M., Cuello E.M., Andorno A.V., Botto E.N. (2014). Primera cita de la Argentina de *Ophelimus maskelli* (Ashmead) (Hymenoptera:Eulophidae) y su parasitoide, *Closterocerus chamaeleon* (Girault) (Hymenoptera:Eulophidae). Rev. Soc. Entomol. Argent..

[24] Wondafrash M., Slippers B., Nambazimana A., Kayumba I., Nibouche S. (2020). Distribution and genetic diversity of five invasive pests of *Eucalyptus* in sub-Saharan Africa. Biol. Invasions.

[25] Lazaro J., Pudjianto, Harahap I.S. (2023). Current Infestation Status and Damage Severity of *Eucalyptus* Gall Wasps, *Leptocybe invasa* (Fisher & La Salle), and *Ophelimus maskelli* Ashmead (Hymenoptera: Eulophidae), Infesting Eucalyptus Germplasms in Tanzania. IOP Conf. Ser. Earth Environ. Sci..

[26] Protasov A., Blumberg D., Brand D., La Salle J., Mendel Z. (2007). Biological control of the gall wasp *Ophelimus maskelli* (Ashmead): Taxonomy and biology of the parasitoid species *Closterocerus chamaeleon* (Girault), with information on its establishment in Israel. Biol. Control.

[27] Sánchez I. (2003). Descubiertas dos nuevas plagas del eucalipto en España. Quercus.

[28] Huber J.T., Mendel Z., Protasov A., La Salle J. (2006). Two New Species of *Stethynium* (Hymenoptera: Mymaridae), Australian Larval Parasitoids of *Ophelimus maskelli* (Ashmead) (Hymenoptera: Eulophidae) on *Eucalyptus*. J. Nat. Hist..

[29] Manuela B., Conceicao B., Nicolas D., Carlos F.J., Zvi M. (2009). Presence of the *Eucalyptus* gall wasp *Ophelimus maskelli* and its parasitoid *Closterocerus chamaeleon* in Portugal: First record, geographic distribution and host preference. Phytoparasitica.

[30] Li H.Y., Huang Y.Y., Huang C.Y., Tang R.T., Huang T.W., Zheng L.X. (2025). Ultrastructure and sense organs of ovipositors in the phytophagous gall-maker pest *Ophelimus maskelli* (Hymenoptera: Eulophidae) and its parasitoid, *Closterocerus chamaeleon* (Hymenoptera: Eulophidae). J. Asia-Pac. Entomol..

[31] Chen H.Y., Yao J.M., Huang S.B., Pang H. (2021). *Ophelimus bipolaris* sp. n. (Hymenoptera, Eulophidae), a new invasive *Eucalyptus* pest and its host plants in China. Insects.

[32] Dong W.X., Zhang Z.N. (2006). Scanning electron microscopy observation of antennae receptors of *Microplitis mediator*. Acta Entomol. Sin..

[33] Liu W.X., Wan F.H. (2007). Scanning electron microscopy observation of antennae of the *Campoletis chlorideae* sensilla. Chin. J. Biol. Control..

[34] Diakova A.V., Makarova A.A., Polilov A.A. (2018). Between extreme simplification and ideal optimization: Antennal sensilla morphology of miniaturized *Megaphragma* wasps (Hymenoptera: Trichogrammatidae). PeerJ.

[35] Hong Q.C., Chen S.Z., Li C.W. (2016). Observation of *Tetrastichus septentrionalis* antenna with scanning electron microscope. Plant Prot..

[36] Onagbola E.O., Fadamiro H.Y. (2008). Scanning electron microscopy studies of antennal sensilla of *Pteromalus cerealellae* (Hymenoptera: Pteromalidae). Micron.

[37] Xu Y., Hong J., Hu C. (2000). Ultrastructural studies on the antennal sensilla of *Pteromalus puparum* (Hymenoptera: Pteromalidae). J. Zhejiang Univ.-Sci..

[38] Li Z., Yang P., Peng Y., Yang D. (2009). Ultrastructure of antennal sensilla of female *Ceratosolen solmsi marchali* (Hymenoptera:Chalcidoidea:Agaonidae: Agaoninae). Can. Entomol..

[39] Zhou D.Y., Zhang L.S., Chen H.Y. (2009). Scanning electron microscopic observation on sensilla of the antenna in female *Diglyphus isaea*. Chin. J. Appl. Entomol..

[40] Zhang Z.F., Liang Q.C., Wu W.J., Huang J. (2007). Ultrastructural Studies on Sensilla of *Quadrastichus erythrinae* Kim (Hymenoptera: Eulophidae) Adult. J. South Chin. Agric. Univ..

[41] Huang F.Y., Xu M., Lin L.L., Liao R.F., Wu Y. (2010). Ultrastructure of sensilla of *Leptocybe invasa*. Chin. J. Appl. Entomol..

[42] Loo J.X., Ong Y.C., Tee S.C., Wong L.W. (2025). Morphometric analysis of antennae of three female Brachymeria species (Hymenoptera: Chalcididae), parasitoids from the oil palm bagworm, *Metisa plana* Walker (Lepidoptera: Psychidae). Int. J. Trop. Insect Sci..

[43] Marco P., Cristina M., Michele M., Chiara S., Milvia C. (2024). Morphological characterization of the antenna of *Torymus sinensis* (Hymenoptera: Torymidae) and a comparison within the superfamily Chalcidoidea. Arthropod Str. Dev..

[44] Popescu E.I., Gostin N.I. (2023). A New Species of *Megastigmus* and First Record of the Genus and Megastigmidae Family from the Paradise of the Maldives Archipelago. Insects.

[45] Popovici O., Bin F., Masner L., Popovici M., Notton D. (2011). *Triteleia peyerimhoffi* comb. n., a remarkably variable circum-Mediterranean scelionid (Hymenoptera, Platygastroidea). ZooKeys.

[46] Chen Y., Wang C., Yu X., Wang B., Liu Z. (2025). A Comparative Morphological Study of the Ultrastructure of Antennal Sensilla in *Sclerodermus guani* (Hymenoptera: Bethylidae). Insects.

[47] Yuan X., Zhang S., Zhang Z., Kong X., Wang H., Shen G., Zhang H. (2013). Antennal morphology and sensilla ultrastructure of the web-spinning sawfly *Acantholyda posticalis* Matsumura (Hymenoptera: Pamphiliidae). Micron.

[48] Zhou T., Huang X., Ullah H., Tang Y., Zhu D., Xu H., Wen Q., Tian X., Tan J. (2024). Comparative SEM Study of Sensilla and Tyloid Structures in the Antennae of Vespinae (Hymenoptera: Vespidae). Insects.

[49] Zacharuk R.Y. (1985). Antennae and sensilla. Comp. Insect Physiol. Biochem. Pharmacol..

[50] Norton W.N., Vinson S.B. (1974). Antennal sensilla of three parasitic Hymenoptera. Int. J. Insect Morphol. Embryol..

[51] Zheng Y.H., Zheng L.X., Liao Y.L., Wu W.J. (2016). Sexual dimorphism in antennal morphology and sensilla ultrastructure of a pupal endoparasitoid *Tetrastichus howardi (Olliff)* (Hymenoptera: Eulophidae). Microsc. Res. Tech..

[52] Ong Y.C., Loo J.X., Tee S.C., Wong W.L. (2024). Distribution and morphometric studies on antennal sensilla of female and male *Pediobius imbreus* (Hymenoptera: Eulophidae). Zoomorphology.

[53] Sang S.W., Siang C.T., Chuan A.P.O., Lim W.W. (2021). Sexual dimorphism of antennal and ovipositor sensilla of *Tetrastichus* sp. (Hymenoptera: Eulophidae). J. Asia-Pac. Entomol..

[54] Mankin R.W., Mayer M.S. (1984). The insect antenna is not a molecular sieve. Experientia.

[55] Ramsey A., Houston T.F., Ball A.D., Goral T., Barclay M.V., Cox J.P. (2015). Towards an understanding of molecule capture by the antennae of male beetles belonging to the genus Rhipicera (Coleoptera, Rhipiceridae). Anat. Rec..

[56] Xu S., Zhang Y.C., Gao S.S., Wang J.S., Zhang K.P. (2021). Ultramorphology of Female *Andricus moriokae* (Hymenoptera: Cynipidae). Sci. Silvae Sin..

[57] Liu X.H., Zhang M., Shi J.N., Li K., Zhang D. (2013). Ultrastructure of antennal sensilla of a parasitoid fly, *Pales pavida* Meigen (Diptera: Tachinidae). Micron.

[58] Sun Z.J., Chen D., Fan X.J., LIu L., Chen Y.J., Zhang C.H., Ren G.W., Liu X.D. (2014). Antennal Ultrastructure of *Aphidius gifuensis* and the Effect of Cold Storage on Antennae. Sci. Agri. Sin..

[59] Schneider D. (1964). The sense of smell in insects. Sci. Am..

[60] Slifer E.H. (1970). Structure of insect sensilla. Annu. Rev. Entomol..

[61] Huang Z.Y., Zhang Y.J., Liu J.Y., Yang Z.D., Lu W., Zheng L.X. (2018). Ultrastructure of female antennal sensilla of an endoparasitoid wasp, *Quadrastichus mendeli* Kim and La Salle (Hymenoptera: Eulophidae: Tetrastichinae). Microsc. Microanal..

[62] Yang P., Li Z.B., Yang D.R., Peng Y.Q., Finn K. (2018). Comparison of the antennal sensilla of females of four fig-wasps associated with *Ficus auriculata*. Acta Oecol..

[63] Wang R., Zhang L., Xu L.L., Zong S.X., Luo Y.Q. (2015). Sensilla on the antennae and ovipositor of the sea buckthorn carpenter moth, *Holcocerus hippophaecolus* Hua et al. (Lepidoptera: Cossidae). Neotrop. Entomol..

[64] Yan S.C., Meng Z.J., Peng L., Liu D. (2011). Antennal sensilla of the pine weevil *Pissodes nitidus* Roel. (Coleoptera: Curculionidae). Microsc. Res. Tech..

[65] Keil T.A. (1999). Morphology and Development of the Peripheral Olfactory Organs. Insect Olfaction.

[66] Gao Y., Luo L.Z., Hammond A. (2007). Antennal morphology, structure and sensilla distribution in *Microplitis pallidipes* (Hymenoptera: Braconidae). Micron.

[67] Van B.J., Boivin G., Bourdais D., Roux O.E. (2007). Antennal sensilla of hymenopteran parasitic wasps: Variations linked to host exploitation behavior. Mod. Res. Educ. Top. Microsc..

[68] Shiota Y., Sakurai T., Daimon T., Mitsuno H., Fujii T., Matsuyama S., Sezutsu H., Ishikawa Y., Kanzaki R. (2018). In vivo functional characterization of pheromone binding protein-1 in the silkmoth *Bombyx mori*. Sci. Rep..

[69] Liu S., Chang H., Liu W., Cui W.C., Liu Y., Wang Y.L., Ren B.Z., Wang G.R. (2020). Essential role for SNMP1 in detection of sex pheromones in *Helicoverpa armigera*. Insect Biochem. Mol. Biol..

[70] Ochieng S.A., Park K.C., Zhu J.W., Baker T.C. (2000). Functional morphology of antennal chemoreceptors of the parasitoid *Microplitis croceipes* (Hymenoptera: Braconidae). Arthropod Struct. Dev..

[71] Ware A., Compton S. (1992). Repeated evolution of elongate multiporous plate sensilla in female fig wasps (Hymenoptera: Agaonidae: Agaoninae). Proc. K. Ned. Akad. Wet..

[72] Aldworth Z.N., Stopfer M. (2012). Olfactory coding: Tagging and tuning odor-activated synapses for memor. Curr. Biol..

[73] Proffit M., Schatz B., Borges R.M., Hossaert-Mckey M. (2007). Chemical mediation and niche partitioning in non-pollinating fig-wasp communities. J. Anim. Ecol..

[74] Segar S.T., Dunn D.W., Darwell C.T., Cook J.M. (2014). How to be a fig wasp, down under: The diversity and structure of an Australian fig wasp community. Acta Oecologica.

